# Unusual Etiology and Presentation for Hyperkalemia—Dialysis Access Recirculation: A Case Report

**DOI:** 10.5811/cpcem.50879

**Published:** 2026-05-25

**Authors:** Joseph L. Kim, Joshua M. Glazer, Amaka Edeani Achufusi, Ryan E. Tsuchida

**Affiliations:** * University of Wisconsin - Madison, School of Medicine and Public Health, Department of Emergency Medicine, Madison, Wisconsin; † University of Wisconsin - Madison, School of Medicine and Public Health, Department of Emergency Medicine, Internal Medicine, and Anesthesiology, Madison, Wisconsin; ‡ University of Wisconsin - Madison, School of Medicine and Public Health, Department of Medicine, Division of Nephrology, Madison, Wisconsin

**Keywords:** hyperkalemia, dialysis, recirculation, case report

## Abstract

**Introduction:**

Hyperkalemia is a common and potentially life-threatening complication of end-stage renal disease, often producing nonspecific symptoms but profound cardiac effects. While nonadherence and dietary indiscretion are typical precipitants, clinicians must also consider the adequacy and effectiveness of dialysis.

**Case Report:**

We report a patient with end-stage renal disease on thrice-weekly hemodialysis who presented with significant bradycardia and altered mental status. Initial prehospital electrocardiogram (ECG) was suspicious for acute coronary syndrome after automated ECG interpretation suggested anterior ST-segment elevation. In the emergency department, the patient was in a junctional escape rhythm with diffuse peaked T-waves. Serum potassium was 7.8 millimoles per liter with concomitant uremia. Despite administration of potassium-shifting therapies bradycardia persisted, and temporary pacing attempts failed. An epinephrine infusion was initiated while arranging for emergent hemodialysis. Following dialysis, the potassium normalized, cardiac conduction returned to sinus rhythm, and the patient’s mental status improved. In the absence of missed dialysis sessions, increased potassium intake, or access site dysfunction, nephrology determined the likely etiology to be dialysis access recirculation from improper cannulation.

**Conclusion:**

Dialysis recirculation is an uncommon but important cause of inadequate clearance leading to life-threatening hyperkalemia. Clinicians should consider this mechanism when confronted with otherwise unexplained electrolyte derangements in compliant dialysis patients.

## INTRODUCTION

In patients with end-stage renal disease, hyperkalemia is a common and dangerous metabolic disturbance. While nonadherence to therapy and dietary indiscretion are frequent contributors, it is also important to consider the adequacy and efficacy of the patient’s dialysis therapy. Dialysis access recirculation is a phenomenon in which dialyzed blood is siphoned into the dialysis circuit instead of the systemic circulation, causing a mixing of dialyzed with undialyzed blood to enter the dialyzer. This leads to poor clearance of electrolytes such as potassium and uremic toxins. While some degree of access recirculation can be expected, complete recirculation is rare and not commonly encountered.

The clinical presentation of hyperkalemia is often subtle and nonspecific, but the associated cardiac consequences may be sudden and lethal. Certain electrocardiogram (ECG) changes such as a widened QRS complex and hyperacute T-waves are classically associated with hyperkalemia, but ECG changes often evolve with the degree of hyperkalemia. Here we present a case of a patient who was adherent to dialysis and presented with an unusual presentation of an uncommon etiology for hyperkalemia. It was ultimately determined to be due to dialysis access recirculation. The patient improved after receiving effective dialysis inpatient.

## CASE REPORT

**Image 1:**
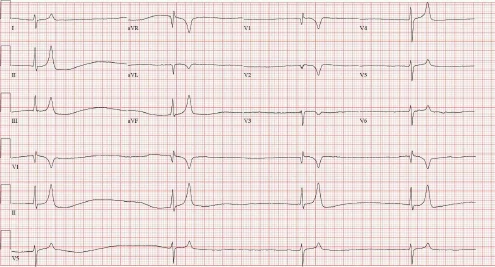
Initial electrocardiogram of patient showing automated machine read for an ST-elevation myocardial infarction.

**Image 2:**
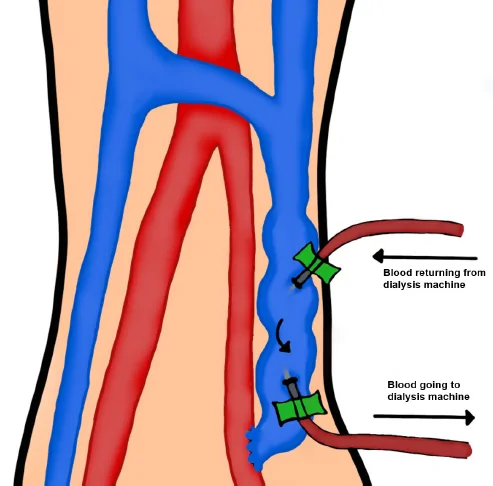
Patient’s electrocardiogram on arrival to the emergency department showing a junctional escape rhythm.

A 56-year-old patient with a past medical history of IgA nephropathy and end-stage renal disease, status post failed kidney transplant, on outpatient hemodialysis three times a week via a left radiocephalic arteriovenous (AV) fistula, called emergency services for chest pain and a sensation of dying. Prehospital responders activated an ST-elevation myocardial infarction (STEMI) alert after the automated ECG report indicated ST-elevations in V4–V6 (Image 1). The patient arrived at the emergency department (ED) somnolent but arousable to painful stimuli. The initial vital signs were significant for bradycardia with a heart rate of 22 beats per minute. The ECG on arrival to the ED revealed bradycardia with a junctional escape rhythm and diffuse peaked T-waves (Image 2).

The initial laboratory studies were significant for potassium, 7.8 millimoles per liter (mmol/L) (reference range: 3.5–5.1 mmol/L); and blood urea nitrogen, o202 milligrams per deciliter (mg/dL) (7–19 mg/dL). Notably, the serum troponin was normal, further lowering the concern for acute coronary syndrome. The hyperkalemia was temporized with insulin, albuterol, and calcium gluconate. Given persistent bradycardia and altered mental status, an epinephrine infusion was initiated. Percutaneous and transvenous pacing were attempted but failed to capture. Nephrology was consulted to initiate emergent dialysis, and the patient was transferred to the intensive care unit (ICU) for further management.

In the ICU, the patient underwent emergent dialysis with a decrease in potassium to 3.5 mmol/L and blood urea nitrogen to 81 mg/dL. The patient’s mental status significantly improved, and bradycardia resolved. On further history-taking and chart review, we found that the patient was entirely adherent to the dialysis schedule with no missed sessions in the prior six months. There were no concerns for increased dietary potassium intake, new infections, nephrotoxin ingestion, or other causes for the elevated potassium and blood urea nitrogen. On further review, the patient’s last fistula-adequacy check via dialysis clearance measurements two weeks prior was normal, and an ultrasound of the fistula while admitted showed patent and normal flow. The case was then discussed with an interventional nephrologist who, based on review of the fistula and skin puncture sites, determined that the hyperkalemia and uremia were likely related to poor cannulation and needle placement. The patient was discharged in stable condition after two full dialysis sessions. The final diagnosis was hyperkalemia secondary to dialysis recirculation.

*CPC-EM Capsule*

What do we already know about this clinical entity?

*Patients on chronic dialysis are at high risk for developing hyperkalemia, and physicians often assess risk by screening for missed dialysis sessions.*

What makes this presentation of disease reportable?

*This case highlights ineffective dialysis clearance as the cause of hyperkalemia resulting from poor needle placement during dialysis.*

What is the major learning point?

*Physicians should include dialysis access recirculation in their differential diagnosis and assess the possibility by examining the fistula and needle-access sites.*

How might this improve emergency medicine practice?

*Assessment of dialysis access sites could lead to quicker diagnosis of hyperkalemia in the setting of an undifferentiated critically ill patient on chronic dialysis.*

## DISCUSSION

Patients on chronic dialysis are at high risk for the development of severe hyperkalemia, which is often attributed to dietary potassium indiscretion and nonadherence to dialysis sessions. As seen here, hyperkalemia can manifest as a multitude of nonspecific symptoms and present with evolving and dynamic ECG findings. Our patient’s ECG initially indicated concerns for acute coronary syndrome with nonspecific ST-segment elevations and hyperacute T-waves with eventual progression toward a junctional escape rhythm. These ECG changes seemed to correlate more with varying degrees of hyperkalemia; however, it was not a consistent representation of actual potassium levels.^1^ A recent meta-analysis of current common practices in the management of hyperkalemia supports the use of intravenous (IV) insulin, IV beta-agonist, and inhaled beta-agonist. No significant benefit was observed with the use of sodium bicarbonate or calcium.^2^ This serves as a good review and understanding of the ongoing discussion on the presentation and management of hyperkalemia in the acute setting.

This case also serves as a reminder of the important factors to evaluate for patients with end-stage renal disease. Physicians will almost always assess for dialysis adherence, but it is also important to evaluate for dialysis adequacy. While factors such as an ineffective dialysis prescription can contribute, we present a case in which the patient’s dialysis was inadequate due to the phenomenon of dialysis access recirculation (Figure). The most common causes of access recirculation are high-grade venous stenosis in the dialysis access, inadequate blood flow rate in the undialyzed side of the dialysis circulation, and improper needle placement by hemodialysis staff.^3^ Improper needle placement is often seen in the form of placing of misdirecting or placing the needles too close together when cannulating the fistula. This ultimately leads to ineffective clearance of the blood. It is important to evaluate the skin and needle placement when evaluating for recirculation.

The National Kidney Foundation’s Kidney Disease Outcome Quality Initiative recommends that the length between needle placement should be at least 3–5 cm to minimize risks of aneurysm formation; however, it does not specify whether this distance decreases recirculation rates. Additionally, it is recommended that the cannulation site be at least 8–10 cm in length, to allow multiple cannulation sites along the fistula and decrease the risk of developing aneurysms, hematomas, and infection.^4^ In our review, we were not able to find any reports of acute severe hyperkalemia attributed to dialysis recirculation from improper AV fistula-needle placement. We did identify one case report of severe recirculation in a mature AV fistula with proven good flow. However, it was attributed to the development of a collateral vessel as opposed to improper needle placement.^5^

In the case presented here, there were no concerns for missed dialysis sessions, anatomical aberrancies, or flow dysfunction. Our patient had also passed an adequacy check about two weeks prior to his presentation and again two weeks after being discharged. After ruling out other common causes, the diagnosis was made of dialysis recirculation due to improper needle placement. To detect recirculation sooner, it is recommended to monitor small solute clearance by measuring urea. Urea is preferred due to its ease of measurement and ability to move freely through the dialyzer membrane. Some studies also suggest that improved dialyzer clearance has been strongly correlated with reduced mortality. Accordingly, the National Kidney Foundation guidelines recommend monthly dialysis adequacy checks via urea clearance monitoring if there are concerns about inadequate dialysis or access dysfunction.^6^ Given this, it is important to consider access recirculation as a possible cause of electrolyte imbalances or other presentations of inadequate dialysis.

## CONCLUSION

This case highlights the manifestations of ineffective dialysis and the sequelae of hyperkalemia. Given that hyperkalemia often presents asymptomatically or with nonspecific symptoms, it is important to be vigilant and suspicious for hyperkalemia in patients on chronic dialysis. While hyperkalemia is classically associated with a wide QRS interval and peaked T-waves, it has variable presentations, including STEMI mimics as well as other arrhythmias. In parallel with evaluating for other causes of otherwise unexplained hyperkalemia, it remains important to assess both dialysis attendance and effectiveness in patients with end-stage renal disease on chronic dialysis. Dialysis recirculation is a risk for all patients on chronic dialysis, and clinicians should be mindful to evaluate for correct needle placement.

## Figures and Tables

**Figure:**
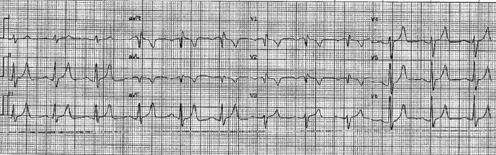
Diagram illustrating dialysis recirculation. Poor needle placement can lead to dialyzed blood being siphoned back into the circuit as opposed to systemic circulation.
